# Physical examination for the detection of sexually transmitted infections among transgender women and travestis in Brazil: acceptability and associated factors

**DOI:** 10.1590/1980-549720240009.supl.1

**Published:** 2024-08-19

**Authors:** Daniel Jason McCartney, Layana Guedes Carvalhal, Camila de Albuquerque Moraes, Philippe Mayaud, Maria Amélia de Sousa Mascena Veras

**Affiliations:** I London School of Hygiene & Tropical Medicine, Faculty of Infectious & Tropical Diseases – London, United Kingdom.; II Secretaria de Estado da Saúde de São Paulo, STD/AIDS Reference and Training Center – São Paulo (SP), Brazil.; III Núcleo de Pesquisa e Direitos Humanos em Saúde da População LGBT+ – São Paulo (SP), Brazil.; IV Santa Casa de São Paulo, School of Medical Sciences – São Paulo (SP), Brazil.

**Keywords:** Transgender, Physical examination, Sexually transmitted infections, Patient preferences, Quality of health care, Brazil

## Abstract

**Objective:**

This study aimed to determine the acceptability and factors associated with uptake of a physical examination for the detection of symptomatic sexually transmitted infections (STIs) by transgender women and *travestis* in Brazil.

**Methods:**

TransOdara was a multi-centric, cross-sectional STI prevalence study conducted among transgender women and *travestis* in five capital cities (Campo Grande, Manaus, Porto Alegre, Salvador and São Paulo) representing all Brazilian regions, between December 2019 and July 2021. A total of 1,317 self-identified transgender women and *travestis* aged ≥18 years were recruited using respondent-driven sampling and responded to a standard questionnaire. A medical consultation was offered including a physical examination and collection of samples from multiple sites to detect various STIs. Factors associated with uptake were investigated by reviewing demographic characteristics of participants who gave permission for physical examination (general, genital, and anorectal).

**Results:**

Most participants (65.4%, 95% confidence interval — 95%CI 62.7–68.0) gave permission for a general examination (including oropharyngeal), with fewer permitting genital (42.3%, 95%CI 39.6–46.0) or anorectal (42.1%, 95%CI 39.4–44.9) examinations. Overall, 34.4% (95%CI 31.8–37.0) of participants refused all examinations. Participants with STI symptoms were significantly more likely to give permission for full examination than asymptomatic participants (64.3 vs. 37.4%, adjusted odds ratio — AOR=3.6, 95%CI 2.4–5.5). Other factors significantly associated with uptake of a full examination in multivariate analysis included age (AOR=1.5 for ≥25 years), religion (AOR=1.7 for Afro-Brazilian, AOR=1.9 for other religions compared to no religion), and education (AOR=2.0 for higher-level).

**Conclusion:**

In the context of STI management, this study found limited acceptance of anogenital examinations among transgender women and *travestis*, with higher acceptance among those with STI symptoms.

## INTRODUCTION

Physical examination remains key to the management of sexually transmitted infections (STIs), along with sexual history taking, to help determine the general status of a patient’s sexual health, to confirm symptoms described by the patient, to identify signs of infection, and to guide any further investigations or treatment required. However, limited evidence is available on the acceptability of physical examination for the detection of STIs among transgender women and *travestis*.

Syndromic management for STIs is based on identifying consistent groups of symptoms and easily recognized signs (i.e., syndromes), and providing treatment that will take care of the most serious pathogens responsible for producing the specific syndrome^1^. Following medical and sexual history taking, the examination typically focuses on the anogenital region and includes a general examination aimed to detect potential manifestations of STIs. Anogenital examinations are inherently intrusive and can be particularly intimidating or unsettling, especially for patients who experience dysphoria with their bodies, experienced past mistreatment from healthcare providers, or experienced violence, including sexual violence, in other contexts^2,3^.

Research conducted in Brazil affirms a high level of exposure to violence, stigma, and discrimination by transgender women, which significantly constrains access to public health and social services^8^.

It is recommended that examinations focus on the current anatomy of the patient and the potential for infection based on the sexual history. Sensitive medical history taking helps to understand individual characteristics in the context of hormone administration and surgical intervention^9^. Consideration should also be given to the detection of other health issues that may not have been previously identified due to limited engagement with health care. For some transgender women, the offer of examination may be welcomed under certain circumstances, as this may be seen as a gender-affirming experience within the healthcare setting^2^.

As transgender women and *travestis* are considered a population at increased risk of STIs and may be reluctant to undergo examination to detect these infections, the objective of this study was to determine the acceptability and factors associated with uptake of a physical examination among participants of the TransOdara study in Brazil.

## METHODS

### Study design and procedures

TransOdara was a multi-centric cross-sectional STI prevalence study conducted among transgender women and *travestis* in five capital cities (Campo Grande, Manaus, Porto Alegre, Salvador, and São Paulo), representing all Brazilian regions. Participants were recruited from December 2019 to July 2021 using respondent-driven sampling (RDS) in each city.

Eligible participants were:

1. Aged 18 years or older;
2. Assigned male sex at birth and self-identified with a feminine gender identity; and
3. Living in the metropolitan area of one of the five capital cities.

An interviewer-led questionnaire collected standard sociodemographic information and responses to questions related to gender-affirming procedures, sexual behaviors, and STI symptoms (including anogenital discharge, ulcers or warts) in the past six months and at study visit. All participants were asked to voluntarily provide biological samples from multiple anatomical sites for testing multiple STIs. Participants could choose whether anorectal, oropharyngeal, or genital samples were self- or provider-collected. For a detailed description of the methodologies used in the TransOdara study, refer to Veras et al.^10^

As part of the study, each participant was asked permission to undergo a physical examination by a study clinician, irrespective of any reported symptoms.

This included independently asking permission to conduct:

1. General examination of the skin, oropharynx, and axillary and groin lymph nodes (to detect possible signs of syphilis, warts, ulcers, inflammation, and adenomegaly);
2. Genital examination (based on the genitalia present to detect the presence of genital discharge, warts, and ulcers): and
3. Anal examination (to detect the presence of anal discharge, warts, and ulcers).

### Data analysis

Study data were collected on standardized case report forms and managed using REDCap electronic data capture tools hosted at the Faculdade de Ciências Médicas da Santa Casa de São Paulo^11,12^. IBM Statistical Package for the Social Sciences — SPSS Statistics for Windows, version 26 (IBM Corp., Armonk, NY, USA) was used for statistical analyses. Demographic and other characteristics of participants who gave permission for each level of examination (general, genital, and anal), and those who permitted examination of all three sites (full examination) were analyzed. Bivariate comparisons were conducted by calculating odds ratios (OR) and 95% confidence intervals (CI) between permission responses and several variables. Variables associated with permission for examination at p*-*values less than 0.1 in the bivariate analyses were included in a multivariate analysis (MVA) using logistic regression to calculate adjusted odds ratios (AOR) and 95%CI for all included variables. Statistical significance was considered for p*-*values less than 0.05 in the MVA.

### Ethical aspects

The project was approved by the Research Ethics Committee of the Santa Casa de Misericórdia de São Paulo (CAAE 05585518.7.0000.5479; opinion n°: 3.126.815 – 30/01/2019), as well as by other participating institutions^10^. Secondary data analysis (by first author) was approved by the London School of Hygiene & Tropical Medicine, UK (Ref: 26700; 14/12/2021). Written informed consent was obtained from all participants.

## RESULTS

### Study population

A total of 1,317 participants aged 18 to 67 years (mean 31.96 years, standard deviation — ±SD 9.86) were recruited from five distinct study locations: Campo Grande (n=181, 13.7%), Manaus (n=339, 25.7%), Porto Alegre (n=192, 14.6%), Salvador (n=202, 15.3%), and São Paulo (n=403, 30.6%). The majority of participants identified as transgender women (56.4%) or ‘*travesti*’ (29.9%), while a smaller proportion identified as women (6.5%) or other gender identities (6.3%). Over one quarter (27.4%) reported undergoing some transition-related surgery or procedure, with a minority (1.7%) reporting neovaginal construction following removal of the penis and scrotum; nearly half (47.6%) were utilizing gender-affirming hormones. Concerning STI symptoms, 21.2% (n=276) reported experiencing symptoms in the previous six months, while 13.1% (n=170) reported symptoms at the study visit, including anogenital warts (n=103), ulcers (n=61), and discharge (n=24).

### Uptake of physical examination

A total of 1,307 records containing examination data (99.2%) were obtained, with 1,297 of these having complete data for all three anatomical sites. Most study participants (65.4%, 95%CI 62.7–68.0) granted permission for a general examination, while a smaller proportion allowed genital examination (42.3%, 95%CI 39.6–46.0) and anal examination (42.1%, 95%CI 39.4–44.9). Overall, less than half (40.6%, 95%CI 37.9–43.4) consented to a comprehensive physical examination (encompassing all three levels). Table 1 presents the uptake for each level of examination according to study location, demonstrating considerable variation. Notably, participants recruited from São Paulo exhibited significantly higher acceptance rates for all examination levels, while those from Manaus displayed the lowest likelihood of granting permission. For instance, participants in São Paulo were 2.9 times (95%CI 2.1–3.9) more likely than those in Manaus to consent to genital examination and 3.2 times (95%CI 2.3–4.3) more likely to consent to anal examination.

**Table 1:** Uptake of physical examination by level and study location among 1,317 transgender women and travestis in Brazil, 2019–2021.

Study location	General		Genital		Anal		Full (all levels)	
	n/N (%)	OR (95%CI)	n/N (%)	OR (95%CI)	n/N (%)	OR (95%CI)	n/N (%)	OR (95%CI)
Manaus	163/338 (48.2)	1.00 (-)	102/338 (30.2)	1.00 (-)	96/333 (28.8)	1.00 (-)	90/333 (27.0)	1.00 (-)
Campo Grande	121/176 (68.8)	2.36 (1.61–3.47)	55/174 (31.6)	1.07 (0.72–1.59)	54/174 (31.0)	1.11 (0.75–1.66)	53/174 (30.5)	1.18 (0.79–1.77)
Salvador	109/202 (54.0)	1.26 (0.89–1.79)	80/202 (39.6)	1.52 (1.05–2.19)	81/202 (40.1)	1.65 (1.14–2.39)	77/202 (38.1)	1.66 (1.15–2.42)
Porto Alegre	115/190 (60.5)	1.65 (1.15–2.36)	93/190 (48.9)	2.22 (1.54–3.20)	91/188 (48.4)	2.32 (1.60–3.36)	90/188 (47.0)	2.48 (1.71–3.61)
São Paulo	346/400 (86.5)	6.88 (4.81–9.84)	222/401 (55.4)	2.87 (2.12–3.89)	225/401 (56.1)	3.16 (2.32–4.30)	217/400 (54.3)	3.20 (2.34–4.37)
Total	854/1306 (65.4)	-	552/1305 (42.3)	-	547/1298 (42.1)	-	527/1297 (40.6)	-

n: number; N: total number; OR: odds ratio; CI: confidence interval.

**Figure 1:**
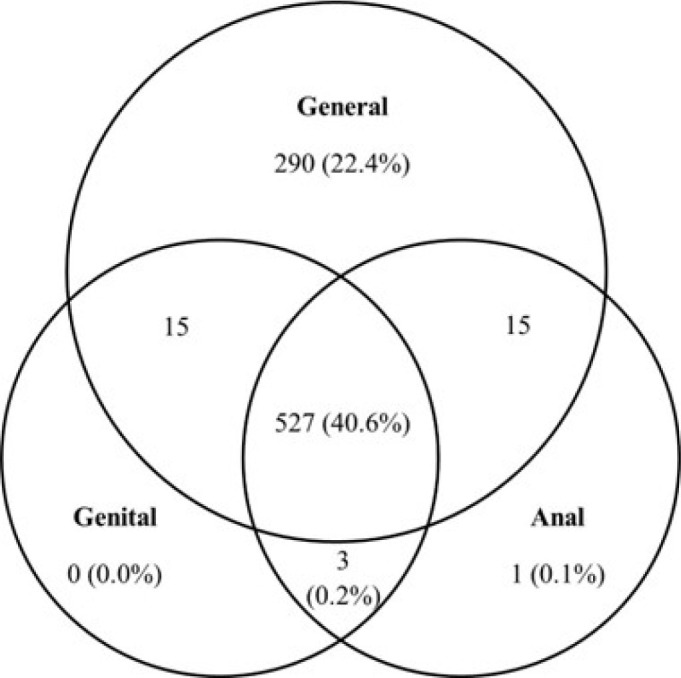
Uptake of general, genital, and anal examinations among study participants with a recorded response at all three levels (n=851), 2019–2021.

Over one third (34.4%, 95%CI 31.8–37.0) of participants declined all examinations. Fewer refused genital and anal examinations exclusively (22.4%, n=290), or any other combinations of refusal: anal only (1.2%, n=15); genital only (1.2%, n=15); general and genital only (0.1%, n=1); general and anal only (0.0%, n=0). Figure 1 illustrates the permissions granted across the three levels of examination. Participants who consented to all three examinations were slightly older (mean 33.12 years, SD±9.94) compared to those who granted permission to some or no examination (mean 31.12 years, SD±9.67). Individuals who reported current STI symptoms at the study visit were most likely to agree to full examinations (64.3%) than those without symptoms (37.4%), with symptomatic participants being less likely to refuse all examinations (13.7%) compared to asymptomatic participants (37.5%).

### Factors associated with uptake at each examination level

#### General examination

Individual-level variables associated with uptake of physical examination are presented in Supplemental Table 1. Apart from study location, the MVA revealed associations with stated religion, education level, use of gender-affirming hormones, and any STI symptoms at study visit. Participants identifying with Afro-Brazilian religion were 1.7 times (95%CI 1.1–2.5) more likely, and those with other stated religions were 1.9 times (95%CI 1.2–3.0) more likely to grant permission compared to those with no stated religion. Higher education levels (post-secondary) were associated with a 2.2 times (95%CI 1.4–3.5) higher likelihood of granting permission compared to lower education levels (none or primary). Participants reporting use of gender-affirming hormones were 1.4 times (95%CI 1.0–1.9) more likely to grant permission than non-users, and those reporting any STI symptoms at the study visit were 4.2 times (95%CI 2.4–7.1) more likely to grant permission than those without symptoms.

#### Genital examination

Individual-level variables associated with uptake of genital examination are presented in Supplemental Table 2. The MVA showed that uptake was associated with age, stated religion, education level, and STI symptoms at the study visit. Participants aged 25 years or older were 1.4 times (95%CI: 1.0–2.0) more likely to grant permission than those aged 18–24 years. Those identifying with Afro-Brazilian religion were 1.6 times (95%CI 1.1–2.4) more likely, and other stated religions were 1.7 times (95%CI 1.2–2.6) more likely to grant permission than those with no stated religion. Higher education levels (post-secondary) were associated with a 1.8 times (95%CI 1.2–2.8) higher likelihood of granting permission compared to those with lower education levels (none or primary). Participants reporting any STI symptoms at the study visit were 4.8 times (95%CI 3.1–7.4) more likely to grant permission. Additionally, uptake of genital examination was less likely among participants identifying with a gender identity other than woman, transgender women, or *travesti*.

#### Anal examination

Individual-level variables associated with uptake of anal examination are presented in Supplemental Table 3. The MVA showed that uptake was associated with age, stated religion, education level, and STI symptoms at study visit. Participants aged 25 years or older were 1.4 times (95%CI 1.0–1.9) more likely to grant permission than those aged 18–24 years. Those identifying with Afro-Brazilian and other stated religions were both 1.6 times (95%CI 1.2–2.2; 1.1–2.3, respectively) more likely to grant permission than those with no stated religion. Higher education levels (post-secondary) were associated with a 2.1 times (95%CI 1.4–3.0) higher likelihood of granting permission compared to lower education levels (none or primary). Participants reporting any STI symptoms at study visit were 3.7 times (95%CI 2.5–5.5) more likely to grant permission.

#### Full physical examination

Individual-level variables associated with uptake of full physical examination (at all three levels) are presented in Table 2 (all variable associations are presented in Supplemental Table 4). The MVA showed that uptake was associated with age, stated religion, education level, and any STI symptoms at study visit. Participants aged 25 years or older were 1.5 times (95%CI 1.0–2.1) more likely to grant permission than those aged 18–24 years. Those identifying with Afro-Brazilian religion were 1.7 times (95%CI 1.2–2.5) more likely, and other stated religions were 1.9 times (95%CI 1.3–2.8) more likely to grant permission than those with no stated religion. Higher education levels (post-secondary) education were associated with a 2.0 times (95%CI 1.3–3.0) higher likelihood to grant permission compared to lower education levels (none or primary). Participants reporting any STI symptoms at the study visit were 3.6 times (95%CI 2.4–5.5) more likely to grant permission for a full examination. It was also observed that uptake of a full physical examination was less likely among participants identifying with a gender identity other than woman, transgender women, or *travesti*.

**Table 2:** Factors significantly associated with uptake of full physical examination (at all three levels) among 1,317 transgender women and travestis in Brazil, 2019–2021.

Variable	n/N (%)	OR (95%CI)	p-value	AOR (95%CI)	p-value
Study location					
Manaus	90/333 (27.0)	1.00 (-)	-	1.00 (-)	-
Campo Grande	53/174 (30.5)	1.18 (0.79–1.77)	0.415	1.72 (1.03–2.88)	0.040
Salvador	77/202 (38.1)	1.66 (1.15–2.42)	0.008	2.10 (1.28–3.45)	0.003
Porto Alegre	90/188 (47.0)	2.48 (1.71–3.61)	<0.001	3.22 (1.91–5.43)	<0.001
São Paulo	217/400 (54.3)	3.20 (2.34–4.37)	<0.001	3.90 (2.52–6.03)	<0.001
Age, years					
18–24	110/344 (32.0)	1.00 (-)	-	1.00 (-)	-
≥25	417/953 (40.9)	1.66 (1.28–2.15)	<0.001	1.47 (1.04–2.08)	0.031
Religion					
No religion	169/470 (36.0)	1.00 (-)	-	1.00 (-)	-
Catholic	118/338 (34.9)	0.96 (0.71–1.28)	0.759	1.26 (0.88–1.80)	0.213
Afro-Brazilian	139/282 (49.3)	1.73 (1.28–2.34)	<0.001	1.72 (1.20–2.49)	0.004
Other*	99/199 (49.7)	1.76 (1.26–2.47)	0.001	1.87 (1.26–2.79)	0.002
Education					
None or primary	121/322 (37.6)	1.00 (-)	-	1.00 (-)	-
Secondary	269/702 (38.3)	1.03 (0.79–1.36)	0.821	1.20 (0.87–1.65)	0.272
Higher-level	135/269 (50.2)	1.67 (1.21–2.33)	0.002	1.98 (1.32–2.97)	0.001
Gender identity					
*Travesti*	150/386 (38.9)	1.00 (-)	-	1.00 (-)	-
Transsexual women	316/767 (41.2)	1.10 (0.86–1.42)	0.445	0.88 (0.64–1.19)	0.396
Woman	49/97 (50.5)	1.61 (1.03–2.51)	0.038	0.99 (0.58–1.67)	0.961
Other identity	11/44 (25.0)	0.52 (0.26–1.07)	0.076	0.13 (0.03–0.67)	0.014
Any STI symptoms at study visit					
No	418/1118 (37.4)	1.00 (-)	-	1.00 (-)	-
Yes	108/168 (64.3)	3.01 (2.15–4.23)	<0.001	3.60 (2.37–5.49)	<0.001

n: number; N: total number; OR: odds ratio; CI: confidence interval; AOR: adjusted odds ratio; STI: sexually transmitted infections.

* Other religion: Evangelical; Judaism; Oriental/Asian; Protestant; Spiritism. Bold indicates p<0.05.

While not significantly associated in the MVA, participants who reported any level of gender-affirmation, including name change on any official documents (OR=1.7, 95%CI 1.3–2.1), or any gender-affirming procedure or surgery (OR=1.5, 95%CI 1.2–1.9), were more likely to grant permission. Among those who reported having a neovagina, over half (54.5%, n=12) permitted a full examination.

## DISCUSSION

This study reports on the acceptability of physical examination for identifying clinical signs of STIs among transgender women and *travestis* in Brazil, a population highly vulnerable to STIs. Although limited evidence indicating that removing the need for examinations in the management of certain STI syndromes (i.e., anorectal discharge) could improve efficiency and cost-effectiveness^13^, global guidelines continue to emphasize the importance of examinations for the comprehensive case management of STIs^1^.

The study findings reveal a varying degree of willingness among participants to undergo specific examination types. While most participants permitted a general examination (65%), fewer permitted a genital (42%) or anal (42%) examination. This lower uptake could potentially lead to incomplete detection of clinical signs of infection. However, notably, the acceptance of physical examination was greater among participants reporting STI symptoms at the study visit. Various factors, including study location, older age, religion, and education, were also found to influence uptake.

Considerable variation of uptake across study locations was evident, with participants in São Paulo being more likely to grant permission. This could be attributed to factors such as the trust built between the São Paulo research team and the transgender community through previous studies. The TransNational study was a longitudinal cohort study to measure HIV incidence of transgender women in São Paulo^14^, with study participants invited to participate in TransOdara. Additionally, as Latin America’s largest city, São Paulo may serve as a hub for those seeking gender-affirming care and a greater sense of community.

Consistent with existing literature, younger participants (aged 18–24) were less likely to grant permission, particularly for genital or anal examinations^15^. In addition, participants with higher education levels demonstrated increased acceptance across all levels of examination. The observed differences by stated religion are not well understood but some possible explanations exist. For example, the higher likelihood of individuals identifying with an Afro-Brazilian religion to grant permission could potentially be explained as these religions tend to be more inclusive of people from sexual and gender minorities^16^. These findings suggest the need for employing approaches with clear, uncomplicated explanations to emphasize the importance of examinations, and those that alleviate potential feelings of shame or embarrassment before or during an examination.

In the bivariate analysis, participants who reported some level of gender-affirmation, such as changing their name on official documents or undergoing gender-affirming procedures or surgery, exhibited a higher likelihood of granting permission for examination. Gender identity also played a role, with those who identify as ‘women’ more likely to permit examination. As reported in the literature, it is possible that a physical examination may be an affirming experience for some transgender women^2^.

Overall, the study noted a limited acceptance of anogenital examinations among transgender women and *travestis*. This underscores the need to establish trust with transgender patients and to refrain from unwarranted examinations. In the context of STI case management, the decision to request specific examinations should be guided by history taking, presenting symptoms, and anatomical relevance for potential infections. For transgender women, self-collected samples for STI screening might be a more suitable and acceptable approach in situations where examinations are unnecessary or refused^17^.

The World Health Organization (WHO) provides guidelines for the management of symptomatic STIs that include simplified flowcharts which involve some level of physical examination^1^. While these guidelines strive to be gender-inclusive (for example, they describe ‘urethral discharge from the penis’ rather than ‘urethral discharge in men’), more specific guidance tailored to and inclusive of transgender and other gender-diverse individuals would be beneficial.

The STI guidelines published by the US Centers for Disease Control and Prevention (CDC) provide specific considerations for transgender and gender diverse persons, including recommendations for creating a welcoming clinical environment and STI screening recommendations^18,19^. Similar gender-affirming guidance is essential within Brazilian clinical settings.

As recommended by the University of California San Francisco (UCSF) Gender Affirming Health Program, a gender-affirming approach to physical examinations is essential. This includes using the correct name and pronouns for patients and employing preferred terms for body parts^9^. Avoiding assumptions about patients’ sexual partners, activities, or risks contributes to a more sensitive approach^3^.

A limitation of this cross-sectional study is the use of RDS for participant recruitment at each study location. This methodology can introduce potential sample and selection bias, requiring results to be interpreted with caution. It is important to note that the findings are not representative of all transgender women and *travestis* in Brazil but reflect the specific networks of the transgender women and *travestis* who were part of the sample at each study location.

In conclusion, the findings from this study reveal a nuanced acceptance of physical examinations among transgender women and *travestis*, particularly noting higher acceptance among those with STI symptoms, which supports its potential application in symptomatic case management. Where not yet established as the standard of care, empowering individuals accessing STI services with options for self-collection of samples (and potentially self-testing) is important, especially for asymptomatic STI screening. To improve examination uptake, it is essential to enhance transgender-specific knowledge among healthcare professionals and integrate a sensitive, gender-affirming approach into clinical guidelines. This approach is vital for public health, as it respects the unique needs of transgender women and *travestis* and promotes more effective and inclusive healthcare services.
