# Supplementary material for: Physical examination for the detection of sexually transmitted infections among transgender women and travestis in Brazil: acceptability and associated factors
Source: Rev Bras Epidemiol. 2024 Aug 19;27(Suppl 1):e240009.supl.1. doi: 10.1590/1980-549720240009.supl.1 (PMC11338536; doi:10.1590/1980-549720240009.supl.1)
Supplement: Supplementary file 1 [file 1980-5497-rbepid-27-suppl1-e240009-s1.pdf]

SUPPLEMENTAL MATERIAL

Supplemental Table 1. Factors associated with uptake of general examination among 1,317 transgender women in Brazil

Variable	n/N (%)	OR (95% CI)	*p*-value	AOR (95% CI)	*p*-value
**Study location**					
Manaus	163/338 (48.2)	1.00 (-)	-	1.00 (-)	-
Campo Grande	121/176 (68.8)	2.36 (1.61-3.47)	**<0.001**	3.07 (1.86-5.07)	**<0.001**
Salvador	109/202 (54.0)	1.26 (0.89-1.79)	0.197	1.76 (1.09-2.82)	**0.020**
Porto Alegre	115/190 (60.5)	1.65 (1.15-2.36)	**0.007**	2.33 (1.37-3.99)	**0.002**
São Paulo	346/400 (86.5)	6.88 (4.81-9.84)	**<0.001**	9.69 (6.11-15.39)	**<0.001**
**Age, years**					
18-24	206/348 (59.2)	1.00 (-)	-	1.00 (-)	-
≥25	648/958 (67.6)	1.44 (1.12-1.86)	**0.005**	1.35 (0.95-1.93)	0.091
**Ethnicity**					
Black	221/347 (63.7)	1.00 (-)	-	1.00 (-)	-
Mixed	374/570 (65.6)	1.01 (0.82-1.44)	0.554	1.22 (0.85-1.76)	0.273
White	228/332 (68.7)	1.25 (0.91-1.72)	0.170	1.16 (0.77-1.74)	0.479
Other[1]	22/45 (48.9)	0.55 (0.29-1.02)	**0.057**	0.54 (0.25-1.18)	0.122
**Religion**					
No religion	295/471 (62.6)	1.00 (-)	-	1.00 (-)	-
Catholic	201/344 (58.4)	0.84 (0.63-1.11)	0.225	1.15 (0.80-1.68)	0.450
Afro-Brazilian	199/282 (70.6)	1.43 (1.04-1.96)	**0.027**	1.68 (1.13-2.51)	**0.011**
Other[2]	155/201 (77.1)	2.01 (1.38-2.94)	**<0.001**	1.87 (1.17-2.98)	**0.009**
**Education**					
None or primary	203/325 (62.5)	1.00 (-)	-	1.00 (-)	-
Secondary	450/706 (38.3)	1.06 (0.81-1.38)	0.692	1.23 (0.88-1.72)	0.220
Higher-level	199/271 (73.4)	1.66 (1.17-2.36)	**0.005**	2.22 (1.41-3.50)	**0.001**
**Housing**					
Temporarily with friends or family	213/341 (62.5)	1.00 (-)	-		
Rents house or apartment	318/476 (66.8)	1.21 (0.90-1.62)	0.200		
Owns house or apartment	227/339 (67.0)	1.22 (0.89-1.67)	0.220		
Other[3]	96/148 (64.9)	1.11 (0.74-1.66)	0.613		
**Gender identity**					
Travesti	246/390 (63.1)	1.00 (-)	-	1.00 (-)	-
Trans woman	511/772 (66.2)	1.15 (0.89-1.48)	0.293	0.97 (0.69-1.34)	0.834
Woman	70/97 (72.2)	1.52 (0.93-2.48)	**0.095**	1.01 (0.55-1.84)	0.981
Other identity	26/44 (59.1)	0.85 (0.45-1.60)	0.605	0.38 (0.13-1.13)	0.082
**Name changed on any official document**					
No	574/923 (62.2)	1.00 (-)	-	1.00 (-)	-
Yes	279/381 (73.2)	1.66 (1.28-2.16)	**<0.001**	1.04 (0.74-1.47)	0.822
**Any gender-affirming procedure**					
No	594/941 (63.1)	1.00 (-)	-	1.00 (-)	-
Yes	257/357 (72.0)	1.50 (1.15-1.96)	**0.003**	0.85 (0.59-1.22)	0.369
**Use of gender-affirming hormones** (current)					
No	363/583 (62.3)	1.00 (-)	-	1.00 (-)	-
Yes	375/534 (70.2)	1.43 (1.11-1.84)	**0.005**	1.41 (1.04-1.92)	**0.027**
**Any STI symptoms in past 6 months**					
No	643/1013 (63.5)	1.00 (-)	-	1.00 (-)	-
Yes	199/275 (72.4)	1.51 (1.12-2.02)	**0.006**	0.96 (0.67-1.39)	0.832
**Any STI symptoms at study visit**					
No	702/1126 (62.3)	1.00 (-)	-	1.00 (-)	-
Yes	146/169 (86.4)	3.83 (2.43-6.05)	**<0.001**	4.15 (2.44-7.05)	**<0.001**

OR: Odds Ratio; **CI**: Confidence Interval; **AOR**: Adjusted Odds Ratio
[1]Other ethnicity: East Asian; Indigenous
[2]Other religion: Evangelical; Judaism; Oriental/Asian; Protestant; Spiritism
[3]Other housing: Any other housing arrangement

Supplemental Table 2. Factors associated with uptake of genital examination among 1,317 transgender women in Brazil

Variable	n/N (%)	OR (95% CI)	*p*-value	AOR (95% CI)	*p*-value
**Study location**					
Manaus	102/338 (30.2)	1.00 (-)	-	1.00 (-)	-
Campo Grande	55/174 (31.6)	1.07 (0.72-1.59)	0.739	1.64 (0.98-2.72)	0.058
Salvador	80/202 (39.6)	1.52 (1.05-2.19)	**0.025**	1.89 (1.16-3.09)	**0.011**
Porto Alegre	93/190 (48.9)	2.22 (1.54-3.20)	**<0.001**	2.94 (1.75-4.92)	**<0.001**
São Paulo	222/401 (55.4)	2.87 (2.12-3.89)	**<0.001**	3.41 (2.21-5.25)	**<0.001**
**Age**, years)					
18-24	117/347 (33.7)	1.00 (-)	-	1.00 (-)	-
≥25	435/958 (45.4)	1.64 (1.27-2.11)	**<0.001**	1.41 (1.00-1.99)	**0.050**
**Ethnicity**					
Black	143/347 (41.2)	1.00 (-)	-		
Mixed	239/570 (41.9)	1.03 (0.79-1.35)	0.830		
White	150/331 (45.3)	1.18 (0.87-1.60)	0.281		
Other[1]	15/45 (33.3)	0.71 (0.37-1.37)	0.312		
**Religion**					
No religion	179/472 (37.9)	1.00 (-)	-	1.00 (-)	-
Catholic	127/343 (37.0)	0.96 (0.72-1.28)	0.794	1.20 (0.84-1.72)	0.310
Afro-Brazilian	143/282 (50.7)	1.68 (1.25-2.27)	**0.001**	1.63 (1.13-2.35)	**0.009**
Other[2]	101/200 (50.5)	1.67 (1.20-2.33)	**0.003**	1.73 (1.17-2.58)	**0.007**
**Education**					
None or primary	129/324 (39.8)	1.00 (-)	-	1.00 (-)	-
Secondary	280/706 (39.7)	0.96 (0.76-1.30)	0.962	1.15 (0.84-1.58)	0.382
Higher-level	141/271 (52.0)	1.64 (1.18-2.27)	**0.003**	1.84 (1.23-2.76)	**0.003**
**Housing**					
Temporarily with friends or family	123/341 (36.1)	1.00 (-)	-	1.00 (-)	-
Rents house or apartment	205/477 (43.0)	1.34 (1.00-1.78)	**0.047**	1.17 (0.81-1.70)	0.399
Owns house or apartment	162/338 (47.9)	1.63 (1.20-2.22)	**0.002**	1.27 (0.86-1.87)	0.234
Other[3]	62/147 (42.2)	1.29 (0.87-1.92)	0.203	0.94 (0.57-1.57)	0.816
**Gender identity**					
Travesti	163/389 (41.9)	1.00 (-)	-	1.00 (-)	-
Trans woman	327/772 (42.4)	1.02 (0.80-1.30)	0.882	0.83 (0.61-1.13)	0.242
Woman	49/97 (50.5)	1.42 (0.91-2.21)	0.127	0.89 (0.52-1.51)	0.887
Other identity	12/44 (27.3)	0.52 (0.26-1.04)	**0.064**	0.12 (0.02-0.61)	**0.011**
**Name changed on any official document**					
No	356/922 (38.6)	1.00 (-)	-	1.00 (-)	-
Yes	195/381 (51.2)	1.67 (1.31-2.12)	**<0.001**	1.23 (0.91-1.66)	0.175
**Any gender-affirming procedure**					
No	374/941 (39.7)	1.00 (-)	-	1.00 (-)	-
Yes	177/356 (49.7)	1.50 (1.17-1.92)	**0.001**	0.91 (0.67-1.25)	0.557
**Use of gender-affirming hormones** (current)					
No	237/582 (40.7)	1.00 (-)	-	1.00 (-)	-
Yes	244/534 (45.7)	1.23 (0.97-1.55)	**0.094**	1.19 (0.89-1.58)	0.240
**Any STI symptoms in past 6 months**					
No	405/1012 (40.0)	1.00 (-)	-	1.00 (-)	-
Yes	142/275 (51.6)	1.60 (1.22-2.09)	**0.001**	0.95 (0.68-1.32)	0.768
**Any STI symptoms at study visit**					
No	432/1124 (38.4)	1.00 (-)	-	1.00 (-)	-
Yes	119/170 (70.0)	3.74 (2.64-5.30)	**<0.001**	4.76 (3.09-7.35)	**<0.001**

OR: Odds Ratio; **CI**: Confidence Interval; **AOR**: Adjusted Odds Ratio
[1]Other ethnicity: East Asian; Indigenous
[2]Other religion: Evangelical; Judaism; Oriental/Asian; Protestant; Spiritism
[3]Other housing: Any other housing arrangement

Supplemental Table 3. Factors associated with uptake of anal examination among 1,317 transgender women in Brazil

**Variable**	n/N (%)	OR (95% CI)	*p*-value	AOR (95% CI)	*p*-value
**Study location**					
Manaus	96/333 (28.8)	1.00 (-)	-	1.00 (-)	-
Campo Grande	54/174 (31.0)	1.11 (0.75-1.66)	0.605	1.30 (0.83-2.05)	0.250
Salvador	81/202 (40.1)	1.65 (1.14-2.39)	**0.007**	1.73 (1.11-2.70)	**0.015**
Porto Alegre	91/188 (48.4)	2.32 (1.60-3.36)	**<0.001**	2.24 (1.42-3.55)	**0.001**
São Paulo	225/401 (56.1)	3.16 (2.32-4.30)	**<0.001**	3.22 (2.18-4.75)	**<0.001**
**Age, years**					
18-24	112/344 (32.6)	1.00 (-)	-	1.00 (-)	-
$\geq$25	435/954 (45.6)	1.74 (1.34-2.25)	**<0.001**	1.39 (1.03-1.88)	**0.032**
**Ethnicity**					
Black	141/345 (40.9)	1.00 (-)	-		
Mixed	232/566 (41.0)	1.01 (0.77-1.32)	0.972		
White	154/330 (46.7)	1.27 (0.93-1.72)	0.129		
Other[1]	15/45 (33.3)	0.72 (0.38-1.39)	0.333		
**Religion**					
No religion	176/471 (37.4)	1.00 (-)	-	1.00 (-)	-
Catholic	125/338 (37.0)	0.98 (0.74-1.31)	0.911	1.13 (0.81-1.58)	0.460
Afro-Brazilian	143/282 (50.7)	1.72 (1.28-2.33)	**<0.001**	1.61 (1.15-2.23)	**0.005**
Other[2]	101/199 (50.8)	1.73 (1.24-2.41)	**0.001**	1.60 (1.10-2.31)	**0.013**
**Education**					
None or primary	127/323 (39.3)	1.00 (-)	-	1.00 (-)	-
Secondary	278/702 (39.6)	1.01 (0.77-1.33)	0.932	1.17 (0.86-1.57)	0.315
Higher-level	140/269 (52.0)	1.68 (1.21-2.32)	**0.002**	2.07 (1.43-2.98)	**<0.001**
**Housing**					
Temporarily with friends or family	126/340 (37.1)	1.00 (-)	-	1.00 (-)	-
Rents house or apartment	201/476 (42.2)	1.24 (0.93-1.65)	0.138	0.92 (0.66-1.28)	0.616
Owns house or apartment	161/335 (48.1)	1.57 (1.16-2.14)	**0.004**	1.10 (0.77-1.57)	0.596
Other[3]	59/145 (40.7)	1.17 (0.78-1.73)	0.451	0.77 (0.49-1.22)	0.260
**Gender identity**					
Travesti	159/386 (41.2)	1.00 (-)	-	1.00 (-)	-
Trans woman	326/768 (42.4)	1.05 (0.82-1.35)	0.683	0.91 (0.69-1.20)	0.505
Woman	50/97 (51.5)	1.52 (0.97-2.37)	**0.067**	1.02 (0.62-1.67)	0.950
Other identity	11/44 (25.0)	0.48 (0.23-0.97)	**0.041**	0.50 (0.23-1.10)	0.086
**Name changed on any official document**					
No	353/917 (38.5)	1.00 (-)	-	1.00 (-)	-
Yes	193/379 (50.9)	1.66 (1.30-2.11)	**<0.001**	1.23 (0.93-1.64)	0.152
**Any gender-affirming procedure**					
No	369/935 (39.5)	1.00 (-)	-	1.00 (-)	-
Yes	177/355 (49.9)	1.53 (1.19-1.95)	**0.001**	0.90 (0.67-1.21)	0.477
**Use of gender-affirming hormones** (current)					
No	238/581 (41.0)	1.00 (-)	-		
Yes	241/531 (45.4)	1.20 (0.94-1.52)	0.137		
**Any STI symptoms in past 6 months**					
No	398/1006 (39.6)	1.00 (-)	-	1.00 (-)	-
Yes	143/274 (52.2)	1.67 (1.28-2.18)	**<0.001**	1.06 (0.78-1.44)	0.717
**Any STI symptoms at study visit**					
No	429/1118 (38.4)	1.00 (-)	-	1.00 (-)	-
Yes	116/169 (68.6)	3.52 (2.49-4.97)	**<0.001**	3.73 (2.53-5.49)	**<0.001**

OR: Odds Ratio; **CI**: Confidence Interval; **AOR**: Adjusted Odds Ratio

[1]Other ethnicity: East Asian; Indigenous

[2]Other religion: Evangelical; Judaism; Oriental/Asian; Protestant; Spiritism

[3]Other housing: Any other housing arrangement

Supplemental Table 4. Factors associated with uptake of full physical examination (at all three levels) among 1,317 transgender women in Brazil

Variable	n/N (%)	OR (95% CI)	*p*-value	AOR (95% CI)	*p*-value
**Study location**					
Manaus	90/333 (27.0)	1.00 (-)	-	1.00 (-)	-
Campo Grande	53/174 (30.5)	1.18 (0.79-1.77)	0.415	1.72 (1.03-2.88)	**0.040**
Salvador	77/202 (38.1)	1.66 (1.15-2.42)	**0.008**	2.10 (1.28-3.45)	**0.003**
Porto Alegre	90/188 (47.0)	2.48 (1.71-3.61)	**<0.001**	3.22 (1.91-5.43)	**<0.001**
São Paulo	217/400 (54.3)	3.20 (2.34-4.37)	**<0.001**	3.90 (2.52-6.03)	**<0.001**
**Age. years**					
18-24	110/344 (32.0)	1.00 (-)	-	1.00 (-)	-
$\geq$25	417/953 (40.9)	1.66 (1.28-2.15)	**<0.001**	1.47 (1.04-2.08)	**0.031**
**Ethnicity**					
Black	135/345 (39.4)	1.00 (-)	-		
Mixed	223/565 (39.5)	1.00 (0.76-1.32)	0.988		
White	148/330 (44.8)	1.25 (0.92-1.70)	0.154		
Other[1]	15/45 (33.3)	0.77 (0.40-1.48)	0.431		
**Religion**					
No religion	169/470 (36.0)	1.00 (-)	-	1.00 (-)	-
Catholic	118/338 (34.9)	0.96 (0.71-1.28)	0.759	1.26 (0.88-1.80)	0.213
Afro-Brazilian	139/282 (49.3)	1.73 (1.28-2.34)	**<0.001**	1.72 (1.20-2.49)	**0.004**
Other[2]	99/199 (49.7)	1.76 (1.26-2.47)	**0.001**	1.87 (1.26-2.79)	**0.002**
**Education**					
None or primary	121/322 (37.6)	1.00 (-)	-	1.00 (-)	-
Secondary	269/702 (38.3)	1.03 (0.79-1.36)	0.821	1.20 (0.87-1.65)	0.272
Higher-level	135/269 (50.2)	1.67 (1.21-2.33)	**0.002**	1.98 (1.32-2.97)	**0.001**
**Housing**					
Temporarily with friends or family	122/340 (35.9)	1.00 (-)	-	1.00 (-)	-
Rents house or apartment	195/475 (41.1)	1.24 (0.93-1.66)	0.136	1.04 (0.72-1.50)	0.851
Owns house or apartment	154/335 (46.0)	1.52 (1.12-2.07)	**0.008**	1.12 (0.76-1.65)	0.582
Other[3]	56/145 (38.6)	1.12 (0.75-1.68)	0.567	0.80 (0.48-1.33)	0.381
**Gender identity**					
Travesti	150/386 (38.9)	1.00 (-)	-	1.00 (-)	-
Trans woman	316/767 (41.2)	1.10 (0.86-1.42)	0.445	0.88 (0.64-1.19)	0.396
Woman	49/97 (50.5)	1.61 (1.03-2.51)	**0.038**	0.99 (0.58-1.67)	0.961
Other identity	11/44 (25.0)	0.52 (0.26-1.07)	**0.076**	0.13 (0.03-0.67)	**0.014**
**Name changed on any official document**					
No	339/916 (37.0)	1.00 (-)	-	1.00 (-)	-
Yes	187/379 (49.3)	1.66 (1.30-2.11)	**<0.001**	1.19 (0.88-1.61)	0.250
**Any gender-affirming procedure**					
No	356/934 (38.1)	1.00 (-)	-	1.00 (-)	-
Yes	170/355 (47.9)	1.49 (1.17-1.91)	**0.001**	0.87 (0.63-1.19)	0.371
**Use of gender-affirming hormones** (current)					
No	227/580 (39.1)	1.00 (-)	-	1.00 (-)	-
Yes	236/531 (44.4)	1.24 (0.98-1.58)	**0.073**	1.15 (0.86-1.53)	0.345
**Any STI symptoms in past 6 months**					
No	385/1005 (38.3)	1.00 (-)	-	1.00 (-)	-
Yes	138/274 (50.4)	1.63 (1.25-2.14)	**<0.001**	1.00 (0.72-1.39)	0.991
**Any STI symptoms at study visit**					
No	418/1118 (37.4)	1.00 (-)	-	1.00 (-)	-
Yes	108/168 (64.3)	3.01 (2.15-4.23)	**<0.001**	3.60 (2.37-5.49)	**<0.001**

OR: Odds Ratio; **CI**: Confidence Interval; **AOR**: Adjusted Odds Ratio
[1]Other ethnicity: East Asian; Indigenous
[2]Other religion: Evangelical; Judaism; Oriental/Asian; Protestant; Spiritism
[3]Other housing: Any other housing arrangement
