# Supplementary material for: Physical examination for the detection of sexually transmitted infections among transgender women and travestis in Brazil: acceptability and associated factors
Source: Rev Bras Epidemiol. 2024 Aug 19;27(Suppl 1):e240009.supl.1. doi: 10.1590/1980-549720240009.supl.1 (PMC11338536; doi:10.1590/1980-549720240009.supl.1)
Supplement: Translation

# Translation: Realização do exame físico para detecção de infecções sexualmente transmissíveis entre mulheres trans e travestis no Brasil

**DOI:** 10.1590/1980-549720240009.supl.1.2

**Authors:** Daniel Jason McCartney, Layana Guedes Carvalhal, Camila de Albuquerque Moraes, Philippe Mayaud, Maria Amélia de Sousa Mascena Veras

**Affiliations:** I London School of Hygiene & Tropical Medicine, Faculty of Infectious & Tropical Diseases – Londres, Reino Unido; II Secretaria de Estado da Saúde de São Paulo, Centro de Referência e Treinamento em DST/Aids – São Paulo (SP), Brasil.; III Núcleo de Pesquisa e Direitos Humanos em Saúde da População LGBT+ – São Paulo (SP), Brasil.; IV Santa Casa de São Paulo, Faculdade de Ciências Médicas – São Paulo (SP), Brasil.

**Keywords:** Mulheres transexuais, Exame físico, Infecções sexualmente transmissíveis, Preferências do paciente, Qualidade da assistência à saúde, Brasil

## Abstract

**Objective:**

Determinar a aceitabilidade e os fatores associados à realização do exame físico para detecção de infecções sexualmente transmissíveis (IST) sintomáticas em mulheres trans e *travestis* no Brasil.

**Métodos:**

Foram utilizados dados do “TransOdara”, estudo transversal de prevalência de IST, realizado em cinco capitais brasileiras (Campo Grande, Manaus, Porto Alegre, Salvador e São Paulo) entre dezembro de 2019 e julho de 2021. As 1.317 mulheres trans e travestis, com idade ≥18 anos, recrutadas por meio do método respondent-driven sampling passaram por entrevistas e responderam a um questionário estruturado. Foi ofertada consulta médica, incluindo realização de exame físico e coleta de amostras de vários locais para detectar diversas IST. Para a análise dos fatores associados à aceitabilidade do exame físico (geral, genital e anorretal), considerou-se as características sociodemográficas das participantes que permitiram a sua realização.

**Resultados:**

A maioria (65,4%; intervalo de confiança – IC95% 62,7–68,0) concedeu permissão para o exame geral (incluindo orofaríngeo), com permissão concedida em menor proporção para os exames genitais (42,3%; IC95% 39,6–46,0) ou anorretais (42,1%; IC95% 39,4–44,9). No geral, 34,4% (IC95% 31,8–37,0) delas recusaram todos os exames. As participantes com sintomas de IST foram significativamente mais propensas a conceder permissão para o exame completo do que as participantes assintomáticas (64,3 vs 37,4%, odds ratio ajustado – AOR=3,6, IC95% 2,4–5,5). Os fatores significativamente associados à aceitabilidade do exame completo na análise multivariada incluíram idade (AOR=1,5 para ≥25 anos), religião (AOR=2,0 para afro-brasileiras, AOR=1,9 para outras religiões em comparação com nenhuma religião) e nível de escolaridade (AOR=2,0 para nível superior).

**Conclusão:**

No contexto do manejo de IST, nós observamos aceitação limitada de exames anogenitais entre mulheres trans e travestis, com maior aceitação entre aquelas com sintomas de IST.

## INTRODUÇÃO

O exame físico é fundamental para o tratamento de infecções sexualmente transmissíveis (IST), juntamente com a obtenção do histórico sexual, para ajudar a determinar o estado geral da saúde sexual de pacientes, confirmar os sintomas descritos por pacientes, identificar sinais de infecção e orientar quaisquer investigações ou tratamentos adicionais necessários. No entanto, existem evidências limitadas sobre a aceitabilidade do exame físico para a detecção de IST entre mulheres trans e travestis.

O manejo sindrômico das IST é baseado na identificação de grupos consistentes de sintomas e sinais facilmente reconhecidos (isto é, síndromes) e na oferta de tratamento que cuidará dos patógenos mais graves responsáveis pela produção da síndrome específica^1^. Após a obtenção do histórico médico e sexual, o exame normalmente concentra-se na região anogenital e inclui um exame geral destinado a detectar possíveis manifestações de IST. Os exames anogenitais são inerentemente invasivos e podem ser particularmente intimidantes ou perturbadores, especialmente para pacientes que sofrem de disforia com seus corpos, que foram maltratados anteriormente por parte de profissionais de saúde ou que sofreram violência, incluindo violência sexual, em outros contextos^2,3^.

Autores de uma pesquisa realizada no Brasil comprovaram um alto nível de exposição à violência, estigma e discriminação por parte de mulheres trans, o que restringe significativamente o acesso à saúde pública e aos serviços sociais^8^.

É recomendado que os exames se concentrem na anatomia atual da paciente e no potencial de infecção com base no histórico sexual. A obtenção sensível do histórico médico ajuda a compreender as características individuais no contexto da administração hormonal e da intervenção cirúrgica^9^. Também deve ser considerada a detecção de outros problemas de saúde que podem não ter sido previamente identificados devido ao acesso limitado com os serviços de saúde. Para algumas mulheres trans, a oferta de exames pode ser bem-vinda em determinadas circunstâncias, pois pode ser vista como uma experiência de afirmação de gênero no contexto dos cuidados de saúde^2^.

Como as mulheres trans e travestis são consideradas uma população com risco aumentado de IST e podem relutar em se submeter a exames para detectar essas infecções, nosso objetivo com este estudo foi determinar a aceitabilidade e os fatores associados à realização de exame físico entre as participantes do estudo TransOdara no Brasil.

## MÉTODOS

### Desenho e procedimentos do estudo

O TransOdara foi um estudo multicêntrico e transversal de prevalência de IST realizado entre mulheres trans e travestis em cinco capitais (Campo Grande, Manaus, Porto Alegre, Salvador e São Paulo), representando todas as regiões brasileiras. As participantes foram recrutadas de dezembro de 2019 a julho de 2021 por meio do método de amostragem *respondent-driven sampling* (RDS) em cada cidade.

As participantes elegíveis foram:

1. Maiores de 18 anos;
2. Sexo masculino atribuído ao nascer e autoidentificação com a identidade de gênero feminina; e
3. Residir na região metropolitana de uma das cinco capitais.

Um questionário padrão formulado pelos investigadores coletou informações sociodemográficas padrão e respostas a perguntas relacionadas a procedimentos de afirmação de gênero, comportamentos sexuais e sintomas de IST (incluindo corrimento anogenital, úlceras ou verrugas) nos últimos seis meses e no momento da consulta do estudo. Todas as participantes foram convidadas a fornecer, voluntariamente, amostras biológicas de vários sítios anatômicos para testar múltiplas IST. As participantes poderiam escolher se as amostras anorretais, orofaríngeas ou genitais seriam autocoletadas ou coletadas pelo prestador de serviço. Para a descrição detalhada das metodologias utilizadas no estudo TransOdara, veja Veras et al.^10^

Como parte do estudo, foi solicitada permissão a cada participante para se submeter a um exame físico por um médico do estudo, independentemente de quaisquer sintomas relatados.

Isso incluiu pedir permissão de forma independente para conduzir:

1. Exame geral da pele, orofaringe e linfonodos axilares e inguinais (para detectar possíveis sinais de sífilis, verrugas, úlceras, inflamações e adenomegalia);
2. Exame genital (com base na genitália presente para detectar a presença de corrimento genital, verrugas e úlceras): e
3. Exame anal (para detectar a presença de secreção anal, verrugas e úlceras).

### Análise de dados

Os dados do estudo foram coletados em formulários padronizados de relato de caso e gerenciados por meio de ferramentas REDCap (Coleta Eletrônica de Dados para Pesquisa), hospedadas na Faculdade de Ciências Médicas da Santa Casa de São Paulo^11,12^. O software *IBM Statistical Package for the Social Sciences — SPSS Statistics for Windows*, versão 26 (IBM Corp., Armonk, NY, EUA) foi utilizado para análises estatísticas. As características demográficas e outros aspectos das participantes que concederam permissão para cada nível de exame (geral, genital e anal) e daquelas que permitiram o exame de todos os três locais (exame completo) foram analisadas. Comparações bivariadas foram realizadas a partir do cálculo de *odds ratio* (razão de chances – OR) e intervalos de confiança (IC) de 95% entre as respostas de permissão e diversas variáveis. As variáveis associadas à permissão para exame com valores de p menores ou p<0,1 nas análises bivariadas foram incluídas em análise multivariada por meio de regressão logística para o cálculo de *odds ratio* ajustado (AOR) e IC95% para todas as variáveis incluídas. Foi considerada significância estatística para valores de p menores que 0,05 ou p<0,05 na análise multivariada.

### Aspectos éticos

O projeto foi aprovado pelo Comitê de Ética em Pesquisa (CEP) da Santa Casa de Misericórdia de São Paulo (CAAE 05585518.7.0000.5479 - Nº parecer: 3.126.815 - 30/01/2019), assim como pelas demais instituições participantes^10^. A análise de dados secundários (pelo primeiro autor) foi aprovada pela London School of Hygiene & Tropical Medicine, Reino Unido (Ref: 26700; 14/12/2021). O consentimento informado por escrito foi obtido de todas as participantes.

## RESULTADOS

### População do estudo

Um total de 1.317 participantes com idades entre 18 e 67 anos (média 31,96 anos, desvio padrão — ±DP 9,86) foram recrutadas em cinco locais distintos do estudo: Campo Grande (n=181, 13,7%), Manaus (n=339, 25,7%), Porto Alegre (n=192, 14,6%), Salvador (n=202, 15,3%) e São Paulo (n=403, 30,6%). A maioria das participantes se identificavam como mulheres trans (56,4%) ou travestis (29,9%), enquanto uma proporção menor se identificou como mulheres (6,5%) ou outras identidades de gênero (6,3%). Mais de um quarto (27,4%) relatou ter sido submetida a alguma cirurgia ou procedimento relacionado à transição, com uma minoria (1,7%) relatando construção de neovagina após a remoção do pênis e do escroto; quase metade (47,6%) utilizava hormônios para afirmação de gênero. Em relação aos sintomas de IST, 21,2% (n=276) relataram ter tido sintomas nos últimos seis meses, enquanto 13,1% (n=170) relataram sintomas na consulta do estudo, incluindo verrugas anogenitais (n=103), úlceras (n=61) e corrimento (n=24).

### Aceitabilidade do exame físico

Foram obtidos 1.307 registros contendo dados de exames (99,2%), sendo 1.297 deles com dados completos dos três locais anatômicos. A maioria das participantes do estudo (65,4%, IC95% 62,7–68,0) concederam permissão para o exame geral, enquanto uma proporção menor permitiu o exame genital (42,3%, IC95% 39,6–46,0) e o exame anal (42,1%, IC95% 39,4–44,9). Em geral, menos da metade (40,6%, IC95% 37,9–43,4) consentiu a realização de um exame físico geral (abrangendo todos os três níveis). Na Tabela 1 apresentamos a aceitabilidade para cada nível de exame de acordo com o local de estudo, demonstrando uma variação considerável. Especialmente, as participantes recrutadas em São Paulo apresentaram taxas de aceitação significativamente mais altas para todos os níveis de exame, enquanto as de Manaus apresentaram a menor probabilidade de conceder permissão. Por exemplo, as participantes de São Paulo tinham 2,9 vezes (IC95% 2,1–3,9) mais probabilidade do que aquelas de Manaus de consentir o exame genital e 3,2 vezes (IC95% 2,3–4,3) mais probabilidade de consentir o exame anal.

**Tabela 1:** Aceitabilidade do exame físico por nível e local do estudo entre 1.317 mulheres trans e travestis no Brasil, 2019–2021.

Local do estudo	Geral		Genital		Anal		Completo (todos os níveis)	
	n/N (%)	OR (IC95%)	n/N (%)	OR (IC95%)	n/N (%)	OR (IC95%)	n/N (%)	OR (IC95%)
Manaus	163/338 (48,2)	1,00 (-)	102/338 (30,2)	1,00 (-)	96/333 (28,8)	1,00 (-)	90/333 (27,0)	1,00 (-)
Campo Grande	121/176 (68,8)	2,36 (1,61–3,47)	55/174 (31,6)	1,07 (0,72–1,59)	54/174 (31,0)	1,11 (0,75–1,66)	53/174 (30,5)	1,18 (0,79–1,77)
Salvador	109/202 (54,0)	1,26 (0,89–1,79)	80/202 (39,6)	1,52 (1,05–2,19)	81/202 (40,1)	1,65 (1,14–2,39)	77/202 (38,1)	1,66 (1,15–2,42)
Porto Alegre	115/190 (60,5)	1,65 (1,15–2,36)	93/190 (48,9)	2,22 (1,54–3,20)	91/188 (48,4)	2,32 (1,60–3,36)	90/188 (47,0)	2,48 (1,71–3,61)
São Paulo	346/400 (86,5)	6,88 (4,81–9,84)	222/401 (55,4)	2,87 (2,12–3,89)	225/401 (56,1)	3,16 (2,32–4,30)	217/400 (54,3)	3,20 (2,34–4,37)
Total	854/1306 (65,4)	-	552/1305 (42,3)	-	547/1298 (42,1)	-	527/1297 (40,6)	-

n: número; N: número total; OR: *odds ratio*; IC: intervalo de confiança.

Mais de um terço (34,4%, IC95% 31,8–37,0) das participantes recusaram todos os exames. Menos participantes recusaram exclusivamente exames genitais e anais (22,4%, n=290), ou qualquer outra combinação de recusa: apenas anal (1,2%, n=15); apenas genital (1,2%, n=15); apenas geral e genital (0,1%, n=1); apenas geral e anal (0,0%, n=0). Na Figura 1 nós ilustramos as permissões concedidas nos três níveis de exame. As participantes que consentiram todos os três exames eram ligeiramente mais velhas (média 33,12 anos, DP±9,94) em comparação com aquelas que concederam permissão para algum ou nenhum exame (média 31,12 anos, DP±9,67). As participantes que relataram sintomas atuais de IST na consulta do estudo tiveram maior probabilidade de concordar com os exames completos (64,3%) do que aquelas sem sintomas (37,4%), sendo as participantes sintomáticas menos propensas a recusar todos os exames (13,7%) em comparação com as participantes assintomáticas (37,5%).

**Figura 1:** Aceitabilidade de exames geral, genital e anal entre as participantes do estudo com resposta registrada em todos os três níveis (n=851), 2019–2021.

### Fatores associados à aceitabilidade em cada nível de exame

#### Exame geral

As variáveis de nível individual associadas à aceitabilidade do exame físico são apresentadas na Tabela Suplementar 1. Além do local do estudo, a análise multivariada demonstrou associações com religião declarada, nível de escolaridade, uso de hormônios para afirmação de gênero e quaisquer sintomas de IST na consulta do estudo. As participantes que se identificaram com religiões afro-brasileiras tiveram 1,7 vezes (IC95% 1,1–2,5) mais probabilidade, e aquelas com outras religiões declaradas tiveram 1,9 vezes (IC95% 1,2–3,0) mais probabilidade de conceder permissão em comparação com aquelas sem religião declarada. Níveis de escolaridade mais elevados (ensino superior) foram associados a uma probabilidade 2,2 vezes (IC95% 1,4–3,5) maior de conceder permissão em comparação com níveis de escolaridade mais baixos (nenhum ou ensino primário). As participantes que relataram o uso de hormônios para afirmação de gênero tiveram 1,4 vezes (IC95% 1,0–1,9) mais probabilidade de conceder permissão do que as não usuárias, e aquelas que relataram quaisquer sintomas de IST na consulta do estudo foram 4,2 vezes (IC95% 2,4–7,1) mais propensas a conceder permissão do que aquelas sem sintomas.

#### Exame genital

As variáveis de nível individual associadas à aceitabilidade do exame genital são apresentadas na Tabela Suplementar 2. De acordo com a análise multivariada, a aceitabilidade estava associada à idade, religião declarada, nível de escolaridade e sintomas de IST na consulta do estudo. Participantes com 25 anos ou mais foram 1,4 vezes (IC95%: 1,0–2,0) mais propensas a conceder permissão do que aquelas com idade entre 18–24 anos. Aquelas que se identificavam com religiões afro-brasileiras tinham 1,6 vezes (IC95% 1,1–2,4) mais probabilidade, e outras religiões declaradas tinham 1,7 vezes (IC95% 1,2–2,6) mais probabilidade de conceder permissão do que aquelas sem religião declarada. Níveis de escolaridade mais elevados (ensino superior) foram associados a uma probabilidade 1,8 vezes (IC95% 1,2–2,8) maior de conceder permissão em comparação com aquelas com níveis de escolaridade mais baixos (nenhum ou ensino primário). As participantes que relataram quaisquer sintomas de IST na consulta do estudo tiveram 4,8 vezes (IC95% 3,1–7,4) mais chances de conceder permissão. Além disso, a aceitabilidade do exame genital foi menos provável entre as participantes que se identificaram com uma identidade de gênero diferente de mulher, mulher trans ou travesti.

#### Exame anal

As variáveis de nível individual associadas à aceitabilidade do exame anal são apresentadas na Tabela Suplementar 3. De acordo com a análise multivariada, a aceitabilidade estava associada à idade, religião declarada, nível de escolaridade e sintomas de IST na consulta do estudo. As participantes com 25 anos ou mais tiveram 1,4 vezes (IC95% 1,0–1,9) mais chances de conceder permissão do que aquelas com 18–24 anos. Aquelas que se identificavam com religiões afro-brasileiras e outras religiões declaradas tinham 1,6 vezes (IC95% 1,2–2,2; 1,1–2,3, respectivamente) mais probabilidade de conceder permissão do que aquelas sem religião declarada. Níveis de escolaridade mais elevados (ensino superior) foram associados a uma probabilidade 2,1 vezes (IC95% 1,4–3,0) maior de conceder permissão em comparação aos níveis de escolaridade mais baixos (nenhum ou ensino primário). As participantes que relataram quaisquer sintomas de IST na consulta do estudo tiveram 3,7 vezes (IC95% 2,5–5,5) mais probabilidade de conceder permissão.

#### Exame físico completo

As variáveis de nível individual associadas à aceitabilidade do exame físico completo (em todos os três níveis) são apresentadas na Tabela 2 (todas as associações de variáveis são apresentadas na Tabela Suplementar 4). Segundo a análise multivariada, a aceitabilidade estava associada à idade, religião declarada, nível de escolaridade e quaisquer sintomas de IST na consulta do estudo. As participantes com 25 anos ou mais tiveram 1,5 vezes (IC95% 1,0–2,1) mais chances de conceder permissão do que aquelas com 18–24 anos. Aquelas que se identificavam com religiões afro-brasileiras tinham 1,7 vezes (IC95% 1,2–2,5) mais probabilidade, e outras religiões declaradas tinham 1,9 vezes (IC95% 1,3–2,8) mais probabilidade de conceder permissão do que aquelas sem religião declarada. Níveis de escolaridade mais elevados (ensino superior) foram associados a uma probabilidade 2,0 vezes (IC95% 1,3–3,0) maior de conceder permissão em comparação com níveis de ensino mais baixos (nenhum ou ensino primário). As participantes que relataram quaisquer sintomas de IST na consulta do estudo tiveram 3,6 vezes (IC95% 2,4–5,5) mais chances de conceder permissão para um exame completo. Também observamos que a aceitabilidade de um exame físico completo era menos provável entre as participantes que se identificavam com uma identidade de gênero diferente de mulher, mulher trans ou travesti.

**Tabela 2:** Fatores significativamente associados à aceitabilidade do exame físico completo (em todos os três níveis) entre 1.317 mulheres trans e travestis no Brasil, 2019–2021.

Variável	n/N (%)	OR (IC95%)	p-valor	AOR (IC 95%)	p-valor
Local do estudo					
Manaus	90/333 (27,0)	1,00 (-)	-	1,00 (-)	-
Campo Grande	53/174 (30,5)	1,18 (0,79–1,77)	0,415	1,72 (1,03–2,88)	0,040
Salvador	77/202 (38,1)	1,66 (1,15–2,42)	0,008	2,10 (1,28–3,45)	0,003
Porto Alegre	90/188 (47,0)	2,48 (1,71–3,61)	<0,001	3,22 (1,91–5,43)	<0,001
São Paulo	217/400 (54,3)	3,20 (2,34–4,37)	<0,001	3,90 (2,52–6,03)	<0,001
Idade (anos)					
18–24	110/344 (32,0)	1,00 (-)	-	1,00 (-)	-
=25	417/953 (40,9)	1,66 (1,28–2,15)	<0,001	1,47 (1,04–2,08)	0,031
Religião					
Sem religião	169/470 (36,0)	1,00 (-)	-	1,00 (-)	-
Católica	118/338 (34,9)	0,96 (0,71–1,28)	0,759	1,26 (0,88–1,80)	0,213
Afro-brasileira	139/282 (49,3)	1,73 (1,28–2,34)	<0,001	1,72 (1,20–2,49)	0,004
Outra*	99/199 (49,7)	1,76 (1,26–2,47)	0,001	1,87 (1,26–2,79)	0,002
Nível de escolaridade					
Nenhum ou ensino primário	121/322 (37,6)	1,00 (-)	-	1,00 (-)	-
Ensino secundário	269/702 (38,3)	1,03 (0,79–1,36)	0,821	1,20 (0,87–1,65)	0,272
Ensino superior	135/269 (50,2)	1,67 (1,21–2,33)	0,002	1,98 (1,32–2,97)	0,001
Identidade de gênero					
Travesti	150/386 (38,9)	1,00 (-)	-	1,00 (-)	-
Mulher transexual	316/767 (41,2)	1,10 (0,86–1,42)	0,445	0,88 (0,64–1,19)	0,396
Mulher	49/97 (50,5)	1,61 (1,03–2,51)	0,038	0,99 (0,58–1,67)	0,961
Outra identidade	11/44 (25,0)	0,52 (0,26–1,07)	0,076	0,13 (0,03–0,67)	0,014
Quaisquer sintomas de IST na consulta do estudo					
Não	418/1118 (37,4)	1,00 (-)	-	1,00 (-)	-
Sim	108/168 (64,3)	3,01 (2,15–4,23)	<0,001	3,60 (2,37–5,49)	<0,001

n: número; N: número total; OR: *odds ratio*; IC: intervalo de confiança; AOR: *odds ratio* ajustado; IST: infecções sexualmente transmissíveis.

* Outra religião: Evangélica; Judaica; Oriental/Asiática; Protestante; Espírita. Negrito indica p<0,05.

Embora não estejam significativamente associados na análise multivariada, as participantes que relataram qualquer nível de afirmação de gênero, incluindo mudança de nome em qualquer documento oficial (OR=1,7, IC95% 1,3–2,1) ou qualquer procedimento ou cirurgia de afirmação de gênero (OR=1,5, IC95% 1,2–1,9), apresentaram maior probabilidade de conceder permissão. Entre aquelas que relataram ter neovagina, mais da metade (54,5%, n=12) permitiu o exame completo.

## DISCUSSÃO

Neste estudo relatamos a aceitabilidade do exame físico para identificação de sinais clínicos de IST entre mulheres trans e travestis no Brasil, uma população altamente vulnerável às IST. Embora existam evidências limitadas indicando que eliminar exames no manejo de IST sindrômica (por exemplo, corrimento anorretal) poderia melhorar a eficiência e custo-efetividade^13^, as diretrizes globais continuam a enfatizar a importância dos exames para o tratamento abrangente de casos de IST^1^.

Os resultados do estudo revelam um grau variável de disposição entre as participantes para se submeterem a tipos específicos de exames. Embora a maioria das participantes tenha permitido o exame geral (65%), poucas permitiram o exame genital (42%) ou anal (42%). Essa menor aceitabilidade tem o potencial de levar à detecção incompleta de sinais clínicos de infecção. Contudo, vale ressaltar que a aceitação do exame físico foi maior entre as participantes que relataram sintomas de IST na consulta do estudo. Vários fatores, incluindo local do estudo, idade avançada, religião e educação, também influenciaram a aceitabilidade.

Foi evidente uma variação considerável na aceitabilidade entre os locais do estudo, sendo as participantes de São Paulo mais propensas a conceder permissão. Isso pode ser atribuído a fatores como a confiança construída entre a equipe de pesquisa de São Paulo e a comunidade trans por meio de estudos anteriores. O estudo TransNational foi um estudo de coorte longitudinal para medir a incidência de HIV em mulheres trans em São Paulo^14^, com participantes do estudo convidadas a participar do TransOdara. Além disso, sendo a maior cidade da América Latina, São Paulo pode servir como um centro para indivíduos que procuram serviços de afirmação de gênero e um sentido maior de comunidade.

Consistente com a literatura existente, as participantes mais jovens (com idades entre 18 e 24 anos) eram menos propensas a conceder permissão, especialmente para exames genitais ou anais^15^. No mais, as participantes com nível de ensino superior demonstraram maior aceitação em todos os níveis de exame. As diferenças observadas por religião declarada não são bem compreendidas, mas existem algumas explicações possíveis. Por exemplo, a maior probabilidade de indivíduos que se identificam com uma religião afro-brasileira de conceder permissão poderia potencialmente ser explicada, uma vez que estas religiões tendem a ser mais inclusivas com pessoas de minorias sexuais e de gênero^16^. Os resultados sugerem a necessidade de empregar abordagens com explicações claras e descomplicadas para enfatizar a importância dos exames e que aliviem potenciais sentimentos de vergonha ou constrangimento antes ou durante um exame.

Na análise bivariada, as participantes que relataram algum nível de afirmação de gênero, como mudança de nome em documentos oficiais ou realização de procedimentos ou cirurgias de afirmação de gênero, apresentaram maior probabilidade de conceder permissão para o exame. A identidade de gênero também desempenhou um papel, sendo que aquelas que se identificam como “mulheres” têm maior probabilidade de permitir o exame. Conforme relatado na literatura, é possível que o exame físico seja uma experiência afirmativa para algumas mulheres trans^2^.

Em geral, observamos uma aceitação limitada para exames anogenitais entre mulheres trans e travestis. É evidente a necessidade de estabelecer confiança com os pacientes trans e de se abster de exames injustificados. No contexto do manejo de casos de IST, a decisão de solicitar exames específicos deve ser orientada pelo histórico, apresentação de sintomas e relevância anatômica para possíveis infecções. Para as mulheres trans, amostras auto-colhidas para o rastreio de IST podem ser uma abordagem mais adequada e aceitável em situações em que os exames são desnecessários ou recusados^17^.

A Organização Mundial da Saúde (OMS) fornece diretrizes para o manejo de IST sintomáticas que incluem fluxogramas simplificados e que envolvem algum nível de exame físico^1^. Embora essas diretrizes se esforcem para serem inclusivas em relação aos gêneros (por exemplo, elas descrevem “secreção uretral do pênis” em vez de “secreção uretral em homens”), seria benéfica uma orientação mais específica, adaptada e inclusiva para indivíduos trans e pessoas com diversidade de gênero.

As diretrizes de IST publicadas pelos Centros de Controle e Prevenção de Doenças (CDC) dos EUA fornecem considerações específicas para pessoas trans e pessoas com diversidade de gênero, incluindo recomendações para a criação de um ambiente clínico acolhedor e recomendações de triagem de IST^18,19^. Orientações de afirmação de gênero semelhantes são essenciais nos ambientes clínicos brasileiros.

Conforme recomendado pelo Programa de Saúde de Afirmação de Gênero (*Gender Affirming Health Program*) da University of California em São Francisco (UCSF), EUA, uma abordagem de afirmação de gênero nos exames físicos é essencial. Isso inclui usar nomes e pronomes corretos para os pacientes e empregar termos preferenciais para partes do corpo^9^. Evitar suposições sobre os parceiros sexuais, atividades ou riscos dos pacientes contribui para uma abordagem mais sensível^3^.

Uma limitação deste estudo transversal é o uso do RDS para recrutamento de participantes em cada local do estudo. Esta metodologia pode introduzir potenciais vieses de amostragem e seleção, exigindo que os resultados sejam interpretados com cautela. Vale ressaltar que os resultados não são representativos de todas as mulheres trans e travestis no Brasil, mas refletem as redes específicas das mulheres trans e travestis que fizeram parte da amostra em cada local do estudo.

Em conclusão, os resultados do nosso estudo revelam uma aceitação diferenciada dos exames físicos entre as mulheres trans e travestis, notando particularmente uma maior aceitação entre aquelas com sintomas de IST, o que corrobora a sua potencial aplicação no manejo de casos sintomáticos. Em casos em que ainda não esteja estabelecido como padrão de cuidados, é importante capacitar os indivíduos usuários de serviços de IST com opções de autocoleta de amostras (e potencialmente autoteste), especialmente para o rastreio de IST assintomáticas. Para melhorar a aceitabilidade dos exames, é essencial melhorar o conhecimento específico das pessoas trans entre os profissionais de saúde e integrar uma abordagem sensível e de afirmação de gênero nas diretrizes clínicas. Esta abordagem é vital para a saúde pública, pois respeita as necessidades únicas das mulheres trans e travestis e promove serviços de saúde mais eficazes e inclusivos.
